# An Unusual Lacerated Tracheal Tube during Le Fort Surgery: Literature Review and Case Report

**DOI:** 10.1155/2016/6298687

**Published:** 2016-11-08

**Authors:** Preeta George, John E. Fiadjoe, Allan F. Simpao

**Affiliations:** ^1^St. Louis Children's Hospital, Washington University, 1 Children's Pl., St. Louis, MO 63108, USA; ^2^Perelman School of Medicine at the University of Pennsylvania and the Children's Hospital of Philadelphia, 3401 Civic Center Blvd., Philadelphia, PA 19104, USA

## Abstract

Maxillofacial surgeries can present unique anesthetic challenges due to potentially complex anatomy and the close proximity of the patient's airway to the surgical field. Damage to the tracheal tube (TT) during maxillofacial surgery may lead to significant airway compromise. We report the management of a patient with a partially severed TT during Le Fort surgery for midfacial hypoplasia and management strategies based on peer-reviewed literature. This case illustrates the clinical clues associated with a damaged TT and explores the challenges of managing this potentially catastrophic issue.

## 1. Introduction

Maxillofacial surgeries can present numerous challenges to anesthesiologists due to the potential for complex facial anatomy and the close proximity of the tracheal tube (TT) to the surgical field. Damage to the TT during maxillofacial surgery can lead to airway compromise; thus, anesthesia providers should have a strategy in place to prevent or mitigate such events. In this case, we report the intraoperative management of a patient with a partially severed nasal TT during a Le Fort surgery.

## 2. Case Description

A 17-year-old, 56 kg male with midface hypoplasia presented for an elective Le Fort-1 advancement surgery with bilateral malar osteotomies. His prior medical history was unremarkable. On physical examination, the patient had a Mallampati-2 airway, and his mental-hyoid distance, mouth opening, and mandibular subluxation were normal. Anesthesia was induced with sevoflurane and oxygen, obtained peripheral IV access, and applied oxymetazoline to both nares prior to smooth nasotracheal intubation with a 6.5 cuffed TT. The TT cuff was inflated with 3 mL of air; auscultation, squeezing of the pilot balloon, and palpation of the patient's neck confirmed the TT cuff's proper inflation and position.

The surgeon placed a throat pack and started the procedure. While performing a left maxillotomy, the surgical team expressed concern that the TT may have been cut because of visible bubbling of gas from the nose after resection of the left lateral nasal wall. The surgeon placed the patient's head in the neutral position while the situation was assessed. The anesthesia team inspected the TT, confirmed that cephalad migration had not occurred, and discovered that the pilot balloon did not sustain inflation. The team called for help, requested additional equipment (difficult airway cart, surgical airway kit), and prepared for a possible reintubation through a bloody field. However, at this point the patient's vital signs, capnograph waveform, and the ventilator's flow-volume loop patterns had all stabilized. After a brief discussion, the intraoperative team agreed to proceed.

Shortly thereafter, when the surgeons turned the patient's head away from the midline neutral position, the anesthesia machine warned of a circuit leak and air bubbles were again observed in the surgical field. The patient's head was immediately returned to the midline position and the leak again disappeared completely. The anesthesia team surmised that the tracheal tube was partially severed, resulting in an aperture that opened when the head was turned away from midline.

**Figure 1:**
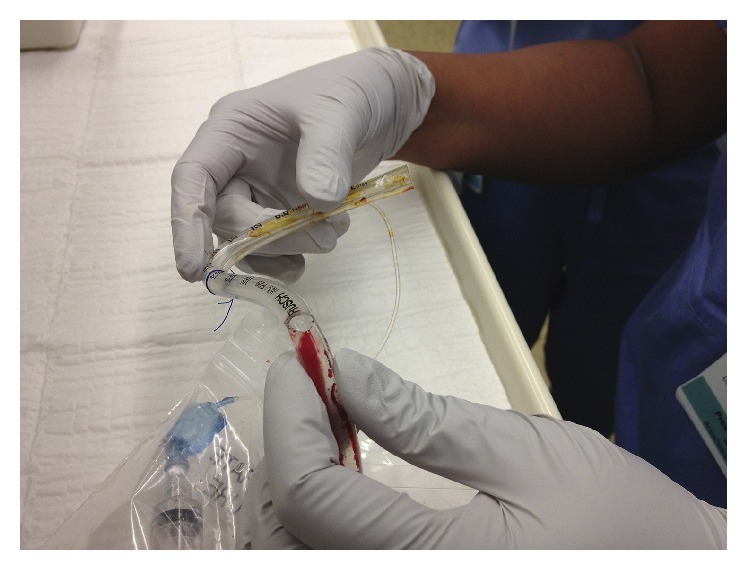
Inspection of the lacerated tracheal tube following the safe emergence and extubation of our patient. Note the widened aperture during bending of the tracheal tube and the severed pilot balloon tubing.

The surgeons stated that the remainder of the case could be performed satisfactorily in the midline position. The patient's ventilation remained completely stable for several minutes, so the decision was made to proceed cautiously with the in situ TT. A tube exchange was eschewed because of the successful conservative measures and the concern of airway loss during an exchange. The remainder of the surgery was uneventful, and we extubated the patient without incident. The patient was transported safely to the postanesthesia care unit where he experienced a full recovery. Inspection of the TT revealed a cut across half of the tube's diameter; the pilot balloon tubing had been severed completely (Figure 1).

## 3. Discussion

Tracheal tube damage during maxillofacial surgery is a potentially catastrophic complication. In some cases, conservative measures such as tube stabilization and laryngeal packing provide adequate ventilatory conditions to complete a surgical case [1, 2]. The definitive solution is to replace the damaged TT, yet reintubation may be difficult due to poor visibility, bleeding, and badly defined tissue planes [3]. Replacing the TT interrupts ventilation, risks aspiration, and can be cumbersome during surgeries of the head, neck, and thorax; furthermore, TT changers and fiberoptic bronchoscopes are not failsafe [4].

Table 1 lists prior reports of damaged TTs during Le Fort procedures as well as the challenges presented by different management strategies. Schwartz et al. described an inability to withdraw the TT more than a few millimeters, where the lacerated end of the tube had formed a barb that caught on a bone snag; their patient was extubated successfully after the lacerated TT was sealed with cement prior to removal [5]. In another case report, a completely severed, wire-reinforced TT obstructed the airway, thereby requiring a surgical airway [6]. Valentine and Kaban reported a case where the pilot tube was severed and the heat from the surgical drill occluded the distal pilot balloon inflation line resulting in a permanently inflated cuff, which complicated the removal of the TT [7].

Anesthesiologists should anticipate TT damage during maxillofacial surgery and take precautionary measures if possible. For a unilateral maxillotomy, intubation via the contralateral nares will reduce the risk of TT damage. The surgeons' use of a nasal septum osteotome with blunt horns may deflect a TT and reduce the likelihood of damage [2]. Intraoperative radiographic imaging may be a useful tool for maxillofacial procedures that require pterygomaxillary disjunction with malar osteotomies. Although this has been neither reported nor studied, this may be a useful guide during the maxillotomy phase of Le Fort surgery to help prevent this complication. The team can then appreciate the proximity of the tracheal tube to the maxilla, and the surgeon can use this information to guide the placement of the osteotomy to avoid TT damage.

If damage to TT occurs during surgery, then a swift assessment of airway patency and ventilation should drive the decision to reestablish the airway. This includes examining the TT depth and auscultating the chest and direct laryngoscopy, if possible [15]. Repositioning the patient's head may improve ventilation in the case of a partially severed tube. Per El-Orbany and Salem [15], a thorough risk/benefit analysis should be performed. Factors that should be considered in this analysis process include the following: (1) length of time for which the patient will require mechanical ventilation; (2) patient's history of a difficult airway or poor laryngeal visualization; (3) the leaked volume and its effect on patient's ventilation; (4) risk of aspiration; (5) tolerance to brief periods of ventilation interruption; (6) expected response to laryngoscopy and intubation; (7) cervical spine status and presence of hard neck collar or halo fixation; and (8) patient's position (supine versus prone or rotated away from anesthesia workstation) [15]. The TT should be exchanged if ventilation and oxygenation are inadequate. Maintaining a sterile field may be challenging; however, a sterile endoscope may allow inspection of the tube prior to exchange if feasible. Emergency airway equipment should be readily available, including a tube exchanger, a video laryngoscope, and a surgical airway kit. The team should prepare for potential difficulty when removing the damaged TT and be ready to perform invasive surgical airway access. A smaller-sized TT can be inserted through a damaged TT to stem a crisis and improve surgical conditions prior to a reintubation attempt [16].

**Figure 2:**
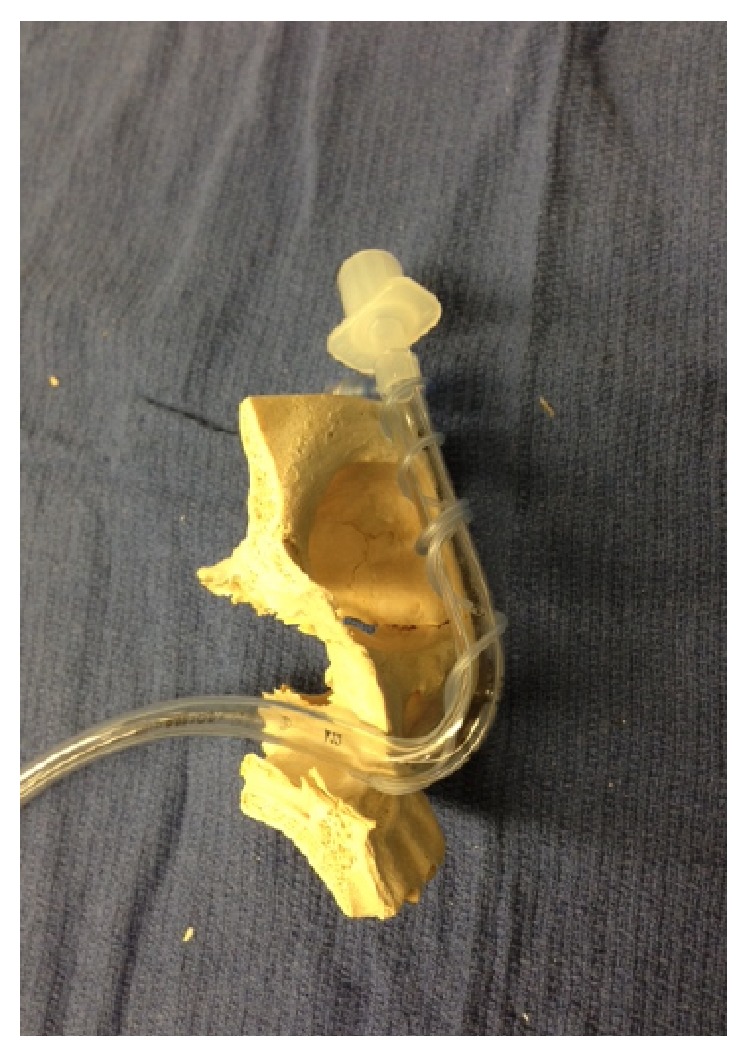
A side and sagittal view of a nasal tracheal tube and its passage through a model of the bony structures of the face.

**Figure 3:**
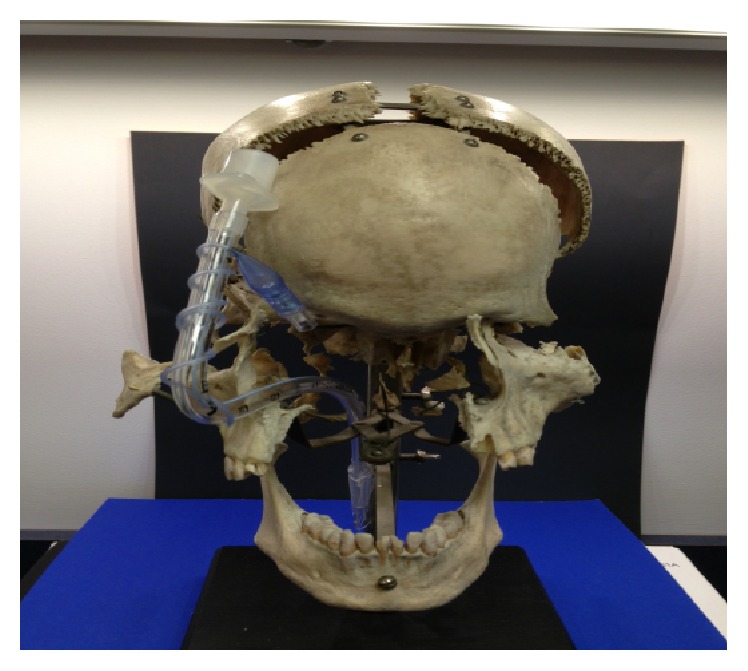
A frontal view of a nasal tracheal tube and its passage through a model of the skull and facial bone structures.

Our case illustrates the challenges of managing a damaged TT midway through a maxillotomy procedure. In our case, we chose to proceed without TT exchange due to adequate oxygenation and ventilation with the head in neutral position. Figure 1 shows the partially severed TT, damaged pilot balloon tubing, and deflated TT cuff from this case. The proximity of the TT to the maxilla in the anterior and lateral views can be observed in Figures 2 and 3. We surmised that the aperture of the severed tube was approximated with the head in neutral positon. Our concerns for a difficult reintubation and the damaged tube catching on bone outweighed the risks of a reasonably stable, albeit suboptimal airway. We remained vigilant throughout the case for any signs of airway compromise and were prepared for a tube exchange and surgical airway placement. Our case illustrates two important clues that should lead anesthesiologists to consider a partially transected TT during Le Fort surgeries: (1) a pilot balloon that fails during the surgery and (2) an intermittent leak that appears and resolves with changes in head position. Either of these signs should prompt immediate investigation of the airway and communication with the surgical team.

## Figures and Tables

**Table 1:** Case reports of damaged tracheal tubes (TTs) during maxillofacial surgery.

Author	Journal/year	Complication	Management
Nair and Balagopal	Indian J Anaesth. 2012 [8]	Partial transection of TT	Unable to ventilate, reintubated over a gum elastic boogie
Ladi and Aphale	Indian J Anaesth. 2011 [6]	Complete TT transection	Flexometallic tube, difficulty removing distal end, emergent tracheostomy
Jain et al.	Indian J Anaesth. 2008 [9]	Partial transection of TT	Unable to ventilate, intubated over a tube exchanger
Bang et al.	Korean J Anesthesiol. 2007 [10]	Partial transection of TT	Continued with a throat pack
Adke and Mendonca	Anaesthesia. 2003 [11]	Partial transection of TT	Noticed after extubation, no leak, intraoperatively
Bidgoli et al.	Eur J Anaesthesiol. 1999 [3]	Partial transection of TT	Unable to ventilate, a nasogastric tube was inserted through the transected TT, which was used as a guide to reintubate
Ketzler and Landers	J Clin Anesth. 1992 [12]	Near total (95%) transection	Continued with a throat pack
Thyme et al.	J Oral Maxillofac Surg. 1992 [13]	Partial transection with pilot tube damage	Unable to ventilate, reintubated, no details
Valentine and Kaban	J Oral Maxillofac Surg. 1992 [7]	Pilot tube damage, unable to deflate cuff	Waited for 2 hrs and for deflation of cuff to extubate
Fagraeus et al.	Anesth Analg. 1980 [14]	Partial transection with pilot tube damage	Unable to deflate cuff, unable to ventilate, aspiration of blood reintubated without difficulty
